# Effects of local and regional climatic fluctuations on dengue outbreaks in southern Taiwan

**DOI:** 10.1371/journal.pone.0178698

**Published:** 2017-06-02

**Authors:** Ting-Wu Chuang, Luis Fernando Chaves, Po-Jiang Chen

**Affiliations:** 1 Department of Molecular Parasitology and Tropical Diseases, School of Medicine, College of Medicine, Taipei Medical University, Taipei, Taiwan; 2 Centro de Investigaciones en Enfermedades Tropicales, Universidad de Costa Rica, San Pedro de Montes de Oca, Costa Rica; 3 Programa de Investigación en Enfermedades Tropicales (PIET), Escuela de Medicina Veterinaria, Universidad Nacional, Heredia, Costa Rica; Johns Hopkins Bloomberg School of Public Health, UNITED STATES

## Abstract

**Background:**

Southern Taiwan has been a hotspot for dengue fever transmission since 1998. During 2014 and 2015, Taiwan experienced unprecedented dengue outbreaks and the causes are poorly understood. This study aims to investigate the influence of regional and local climate conditions on the incidence of dengue fever in Taiwan, as well as to develop a climate-based model for future forecasting.

**Methodology/Principle findings:**

Historical time-series data on dengue outbreaks in southern Taiwan from 1998 to 2015 were investigated. Local climate variables were analyzed using a distributed lag non-linear model (DLNM), and the model of best fit was used to predict dengue incidence between 2013 and 2015. The cross-wavelet coherence approach was used to evaluate the regional El Niño Southern Oscillation (ENSO) and Indian Ocean Dipole (IOD) effects on dengue incidence and local climate variables. The DLNM results highlighted the important non-linear and lag effects of minimum temperature and precipitation. Minimum temperature above 23°C or below 17°C can increase dengue incidence rate with lag effects of 10 to 15 weeks. Moderate to high precipitation can increase dengue incidence rates with a lag of 10 or 20 weeks. The model of best fit successfully predicted dengue transmission between 2013 and 2015. The prediction accuracy ranged from 0.7 to 0.9, depending on the number of weeks ahead of the prediction. ENSO and IOD were associated with nonstationary inter-annual patterns of dengue transmission. IOD had a greater impact on the seasonality of local climate conditions.

**Conclusions/Significance:**

Our findings suggest that dengue transmission can be affected by regional and local climatic fluctuations in southern Taiwan. The climate-based model developed in this study can provide important information for dengue early warning systems in Taiwan. Local climate conditions might be influenced by ENSO and IOD, to result in unusual dengue outbreaks.

## Introduction

Dengue Fever (DF) is an important vector-borne disease that is mainly transmitted in tropical and subtropical regions by several *Aedes* species of mosquito. *Aedes aegypti* (Linnaeus) is considered the main vector for dengue transmission in urban regions, and *Aedes albopictus* (Skuse) is considered the secondary and more rural vector [1]. The incidence of dengue is increasing, and has reached an estimated 390 million annual cases, of which 96 million present with clinical symptoms [2, 3]. Severe dengue infection, mediated by an antibody-dependent enhancement (ADE) mechanism, can cause hemorrhagic fever, shock syndrome, and even death if patients do not receive appropriate medical care [4]. The changing geographical distribution and increasing burden of dengue epidemics in recent years could be related to ongoing climate change, frequent global travel activity, or unplanned urbanization [5–7]. The concurrent circulation of different serotypes also increases the risk of severe clinical outcomes and is a huge concern for public health.

Climate variability is a major driver affecting dengue occurrence that has been widely discussed in the previous literature [8–12]. In Vietnam, dengue risk is associated with temperature, humidity, and precipitation, with different lag windows [13]. In Thailand, dengue transmission increases with monthly minimum temperature but decreases with precipitation in the previous two months [14]. Higher absolute humidity and mean temperature are associated with dengue transmission but vary across different periods of time in Singapore [15]. The biological mechanism of dengue and climate is mediated through the impact of various climate conditions on mosquito populations, virus propagation, and vector-host interaction [2]. Higher temperatures could alter vectorial capacity by reducing the duration of mosquito development and the extrinsic incubation period (EIP) of the virus inside mosquito vectors [16, 17]. Precipitation could provide water for breeding because *Aedes* spp. mosquitos prefer artificial containers around human-made environments [10]. However, mosquito survival is also reduced by extreme temperatures and large rainfall events, and the non-linear relationships should not be ignored [11, 18]. The El Niño Southern Oscillation (ENSO) effect and its impact on dengue transmission and local weather conditions has also been discussed elsewhere [19–23]. Dengue prevention still relies on effective vector control, source reduction, and community-based health education, as there is no specific treatment and the commercial dengue vaccine is only available in a few countries [24]. The concept of a dengue early warning system (DEWS) has been proposed in many studies as a way of enhancing control and prevention efficacy for policy makers [25–27]. Climate-based models play an important role in forecasting dengue incidence in such systems [26, 28–30].

Taiwan, located at the northern edge of Southeast Asia, has experienced annual dengue outbreaks since 1980 [31]. Unlike other Southeast Asian countries where dengue is endemic, the dengue virus is likely imported into Taiwan by international travelers each spring, causing epidemics during the subsequent summer and fall [31]. All four serotypes of dengue virus have been reported in Taiwan since 1981. Although the imported cases play an important role in introducing the dengue virus to Taiwan, the number of imported cases is not a good predictor of the size of the subsequent outbreak, given the high proportion of asymptomatic infections [32]. Thus, the scale of dengue outbreaks may be more related to climatic fluctuations and vector control activities. Associations between temperature, relative humidity, precipitation and dengue transmission have been mentioned in previous studies conducted in Taiwan but no consistent associations have been found and no prediction model has been developed for dengue early warning system [32–36].

Taiwan experienced unprecedented dengue outbreaks in 2014 and 2015, and it remains unclear whether these large outbreaks were associated with climatic fluctuations. The current strategies of vector control and disease prevention are only initiated after identifying the first locally acquired human case. This approach makes it difficult to control the propagation of the vector and virus in the early stages. Thus, developing climate-based forecasting models would make it possible to deliver an early warning message to public health workers to enable them to promptly launch control or prevention activities. For these reasons, this study aims to explore the associations between dengue transmission and climatic factors in southern Taiwan, using long-term epidemiological surveillance data. Our hypothesis is that transmission is influenced by inter-annual climate variations that affect mosquito ecology and subsequent dengue virus transmission. We analyzed both local and regional climate phenomena using a distributed non-linear model and wavelet coherent approach. The best fitting model with local climate parameters was used to forecast most recent dengue outbreaks in southern Taiwan.

## Methods

### Study area

**Fig 1:**
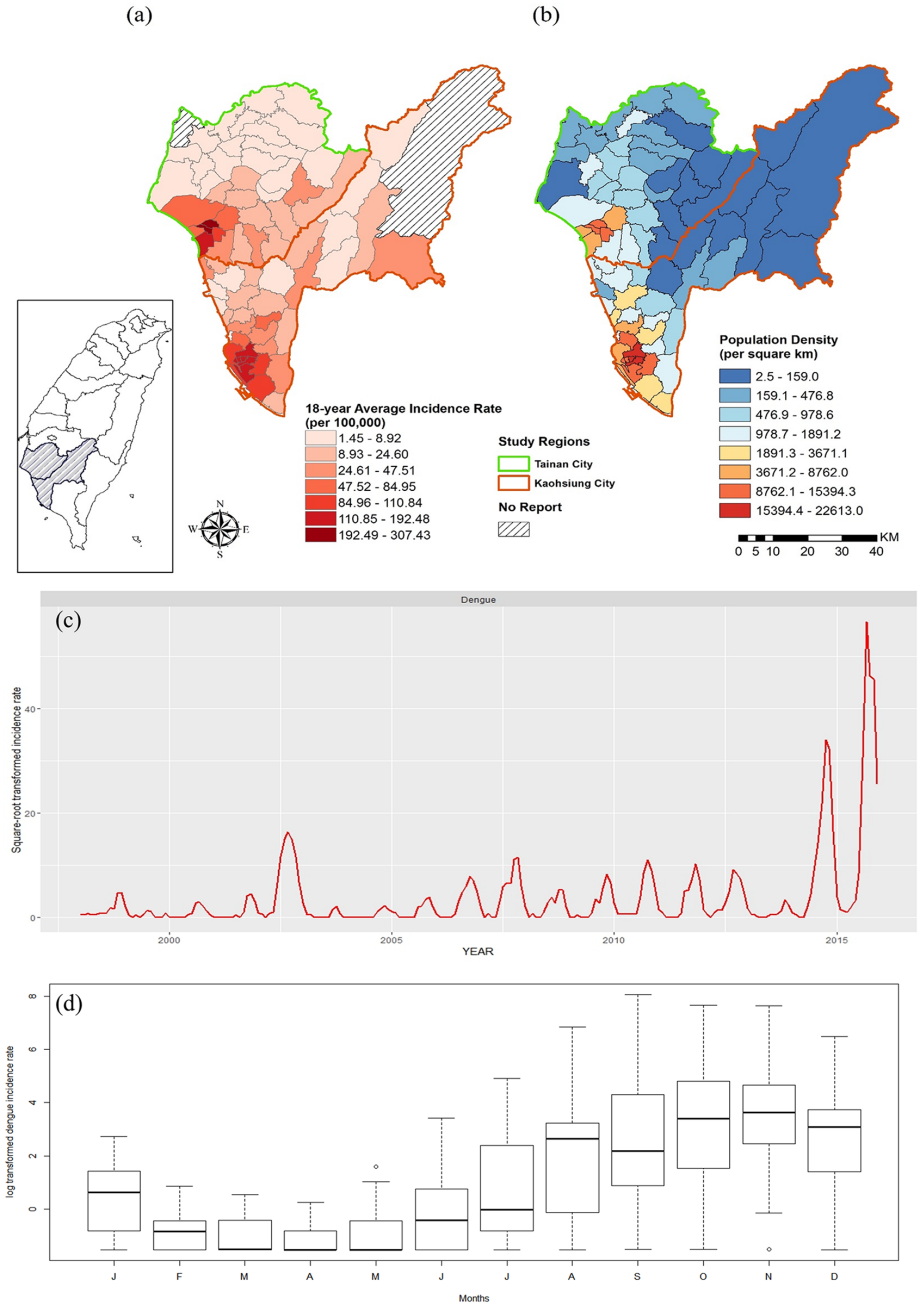
(a) The 18-year average dengue incidence rates in southern Taiwan, by district, from 1998 to 2015. District color indicates dengue incidence rate. (b) The district-level population density in southern Taiwan. (c) Monthly dengue incidence rates in the study area from 1998 to 2015. (d) Boxplot of the 18-year monthly average dengue incidence rates from 1998 to 2015. The solid black indicates the median value. The ends of the whiskers are maximum and minimum values and the circle indicates the outlier.

This study centers on two metropolitan dengue hotspots in Taiwan, which account for 90% of all dengue cases in Taiwan: Kaohsiung City (2,951.85 km^2^) and Tainan City (860.29 km^2^) (Fig 1a). Both cities are in southwestern Taiwan and within the tropical climate zone where *Ae*. *aegypti* and *Ae*. *albopictus* co-exist in urban and peri-urban regions. The mean temperature range is 17.6–29.2°C and the annual average precipitation is approximately 2,000 mm. The main rainy season is between June and September, and the “plum rains” (also referred to as the East Asia rainy season) in May and June bring continuous rainfall and result in high humidity. Precipitation fluctuations in July and August strongly depending on the number of typhoons, which may also influence dengue transmission [37]. The population is highly clustered in these two metropolitan areas with high dengue incidence (Fig 1a and 1b). For the subsequent analyses, we combined the data from the two cities, given their spatial proximity and similarities in terms of climate patterns and urban structure.

### Dengue cases

Dengue fever is a category 2 notifiable infectious disease in Taiwan, and must be reported to the Taiwan Center for Disease Control (CDC) by physicians within 24 hours of identifying a suspected case. Suspected dengue cases are confirmed by the Taiwan CDC based on the presence of anti-dengue IgM, nucleotide sequence, or viral isolation. Since 2014, nonstructural protein 1 (NS1) antigen detection has been used as a rapid diagnostic technique in Taiwan. The patient’s travel history is also obtained to clarify whether the case was imported or locally acquired. Only indigenous cases retrieved from the National Notifiable Disease Reporting System between 1998 and 2015 are included in the study.

The weekly summaries were used to analyze dengue transmission and local climate parameters, and the monthly summaries were used to analyze regional climate conditions. Annual population estimates were obtained from the Taiwan Ministry of the Interior. The population estimates were used as a denominator to estimate dengue incidence rates (per 100,000 population). During the study period, the population in the study area gradually increased from 4,511,476 to 4,664,459. The research protocol was approved by the Taipei Medical University-Joint Institutional Review Board (No. 201308015), and all data were analyzed anonymously.

### Climate data

Meteorological data were acquired from two weather stations (KS: 22°34’ N, 120°18’ E and TN: 23°02’ N, 120°13’ E) near the Kaohsiung and Tainan metropolitan regions. Given the close spatial proximity of the two cities, we averaged the data from the two weather stations throughout the study period. The weekly and monthly data for multiple climate parameters were averaged from the daily data, including mean temperature, maximum temperature, minimum temperature, precipitation, and relative humidity. Because the three temperature measurements are highly correlated, only minimum temperature is included in the analysis, which was considered a more important index in the previous study [35].

In addition to the local climatic fluctuations, the impact of regional climate phenomena (i.e. the El Niño Southern Oscillation and Indian Ocean Dipole) on dengue transmission were investigated using the monthly data. ENSO events were usually evaluated using sea surface temperature (SST) anomalies in the equatorial Pacific Ocean [38]. Since there are various indices that can be used to characterize ENSO events, we evaluated the multivariate ENSO index (MEI), Niño anomaly index at region 1+2, region 3.4, region 3, and region 4 to check which index was more closely related to dengue outbreaks in southern Taiwan (S1 Fig). The Niño 3.4 region (from 170°W to 120°W) was used in our analysis because of its high coherence with major dengue outbreaks in the study area. The monthly scale for the ENSO indices was obtained from the Earth System Research Laboratory of the National Oceanic and Atmospheric Administration (NOAA). The IOD effect was evaluated using the dipole mode index (DMI), which measures SST anomalies in the Indian Ocean [39]. Previous studies showed that IOD could be associated with malaria transmission risk and local precipitation in highland areas of eastern Africa [40, 41]. Our previous work also showed IOD to have an influence on dengue outbreaks in Pakistan [42]. Thus, we would like to understand the impact of IOD on dengue transmission in Taiwan. The monthly DMI was obtained from the Japan Agency for Marine-Earth Science and Technology (JAMSTEC).

### Statistical analysis

We performed a couple of analyses to assess: (1) the effect of local climate variations on dengue incidence in southern Taiwan (weekly data) and the predictive performance of the model of best fit; (2) the association between regional climate phenomena (ENSO and IOD), dengue inter-annual outbreaks, and local climate parameters (monthly data).

### Local climate variability

The distributed lag non-linear model (DLNM) examines non-linear relationships and addresses multicollinearity issues by applying spline smoothing techniques or polynomial functions. It can handle lag effects and non-linear relationships simultaneously using a bi-dimensional function. The delayed effect is important in dengue transmission and climate fluctuations, which is related to the duration of the mosquito life cycle and virus propagation [23, 43]. The growth of mosquito abundance might be related to the higher temperature or precipitation a few weeks or months previously. This approach has been used in many studies to evaluate non-linear relationships between climate factors and mosquito-borne diseases [15, 43, 44]. The formula of the DLNM is shown below:

$$Y_{t}=linear\;model\;\left(t=1,2,3\ldots ,n\right)$$ (1)

$$\mu_{t}=\;\alpha +\sum_{j=1}^{5}\beta_{s}\left(y_{t-j}\right)+\sum_{h=1}^{H}\beta_{k}\left(X_{t,h}\right)++\;\varepsilon_{t}$$ (2)

We used a linear regression model combined with an autoregressive term ($\sum_{j=1}^{5}\beta_{s}\left(y_{t-j}\right)$) to account for seasonal trends and autocorrelation. *Y*_*t*_ denotes the weekly dengue incidence rate (per 100,000) and *μ*_*t*_ is the expected weekly dengue incidence rate (per 100,000) at week *t*. *X*_*t*,*h*_ represents the weekly climate variables at week *t* and lag week *h*. *H* is the maximum lag (24 weeks) in the analysis. Dengue incidence exhibits seasonality and strong autocorrelation (S2 Fig). We compared different techniques to adjust for these effects in the model, and the autoregressive term (lag = 5) demonstrated better results in terms of adjusting for seasonality and autocorrelation than other methods (S1 Table; S3 Fig). *α*, *β*_*s*_, *β*_*k*_, and *ε*_*t*_ represent the intercept, coefficients of autoregressive terms, coefficients of covariates, and the error term. In this analysis, the natural cubic spline function was applied to climate variables (minimum temperature, precipitation, and relative humidity) and the polynomial function was applied to lag effects. The degrees of freedom (df) for the spline and polynomial functions were selected using a nested loop that searched for the minimum Akaike Information Criterion (AIC) by systematically searching over a gradient that explored df for the spline ranging from 1 to 20, and df for the polynomial function ranging from 1 to 10. We chose the AIC as a model selection criterion given that our dengue time series was very long, and that the metric has a simple interpretation where a model is chosen when the AIC, a trade-off function between number of parameters and goodness of fit, is minimized [45]. The relative importance of the climate variables was compared using AIC differences. Exposure-response analyses were performed to examine the effects of each variable under specific values and lags. To validate the predictive performance, models were developed using data from 1998 to 2012 as training data. Forecasts for 2013, 2014 and 2015 were then generated with the best fit model and compared against the observed data. We focused on the best fit model given that prior research has shown best fit models produce more accurate forecasts [46–49]. More specifically, we developed an algorithm that used the fitted coefficients and climatic covariates with five weeks of lag (i.e., between t-4 and t-23) and weekly dengue incidence from the five previous weeks (i.e., from t to t-4) to forecast incidence at time t+1. For forecasts at time t+2, we updated the time windows for the climatic covariates to account for five weeks of lag (i.e., used data between t-3 and t-22) and we then employed the incidence forecasted for time t+1 (as the first autoregressive lag) and dengue incidence data from t to t-3 (for the second to fifth autoregressive lags) to have data for the 5 autoregressive lags in the model. More generally, we have that in a forecast for n (as long as 1≤n≤5) time steps ahead, the time window for climatic covariates will slide between t-5+n and t-24+n, and will use dengue observations between t-5+n (fifth autoregressive lag) and t (for the n autoregressive lag) and, while n>1, forecasts between t+n-1 (for the first lag) and t+1 (for the n-1 autoregressive lag). Given the time lag of the climatic covariates no climatic predictions are required for n≤5 weeks, and for n>5 all autoregressive predictors are based on previous dengue incidence forecasts. We, therefore set “n” from 1 to 6 weeks in order to understand forecasting accuracy over different time windows. We included 6 weeks to gain insights about the prediction ability of the model when all autoregressive components are themselves based on forecasts, and assuming inputs from perfect climatic forecasts, something that was possible given the retrospective nature of our analysis, which allowed us to knew the value of the climatic covariates for n = 6. The predictive accuracy was then evaluated using the predictive R-squared, which has been applied in previous studies on early warning systems of disease transmission, and which measures a model forecasting ability by comparing the variance of residuals with the observed data variance [49]. The predictive R-squared is estimated as 1–(mean square error/variance of the series) and the interpretation is similar to the R-squared in the linear regression model [50]. The DLNM was performed using R software (version 3.3.2) with the “dlnm.”

### Regional climate phenomena

Regional climate patterns usually exhibit non-stationary characteristics that cannot be captured using a stationary time-series model [20]. For instance, ENSO usually exhibits a 2-to-7-year cycle, although uncertainty can make the cycle unpredictable [51]. Wavelet analysis provides a useful tool for handling these non-stationary structures and transient relationships. The theoretical details and applications of wavelet analysis have been described in previous studies [52, 53]. We performed cross-wavelet coherent analyses using R software (version 3.3.2) with the “biwavelet” package to evaluate the effects of ENSO and IOD on dengue transmission and local climate variables.

## Results

A total of 71,793 dengue cases were reported in southern Taiwan between 1998 and 2015, and this area experienced multiple dengue outbreaks, especially in 2014 and 2015 [The number of cases were 15,155 and 42,482] (Fig 1c). Dengue transmission became more active after 2006. Transmission usually starts in June and reaches its peak in October and November, indicating strong seasonality (Fig 1d).

**Fig 2:**
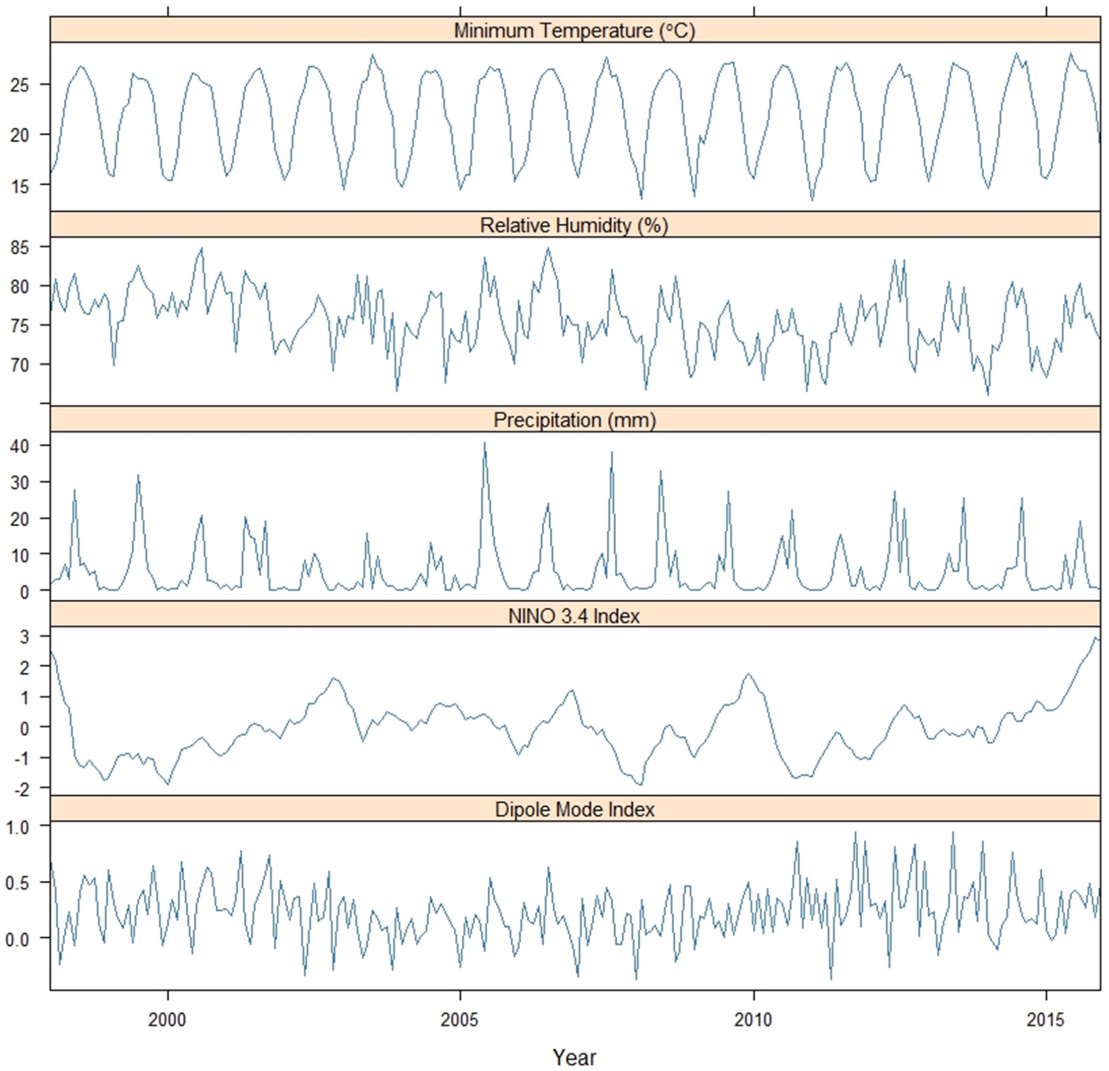
Monthly climate parameters in southern Taiwan from 1998 to 2015.

The monthly meteorological parameters during the study period are summarized in Fig 2. Minimum temperature exhibited relatively stable seasonality, and the relative humidity and precipitation exhibited inter-annual variations that were higher during the spring and summer rainy season. The monthly Niño 3.4 index exhibited a significantly increasing pattern in 2015 with inter-annual fluctuations. DMI varied with no clear pattern. The highest annual minimum temperatures during the study period were observed in 2015, which might be related to the strong ENSO event that year (S2 Table).

**Fig 3:**
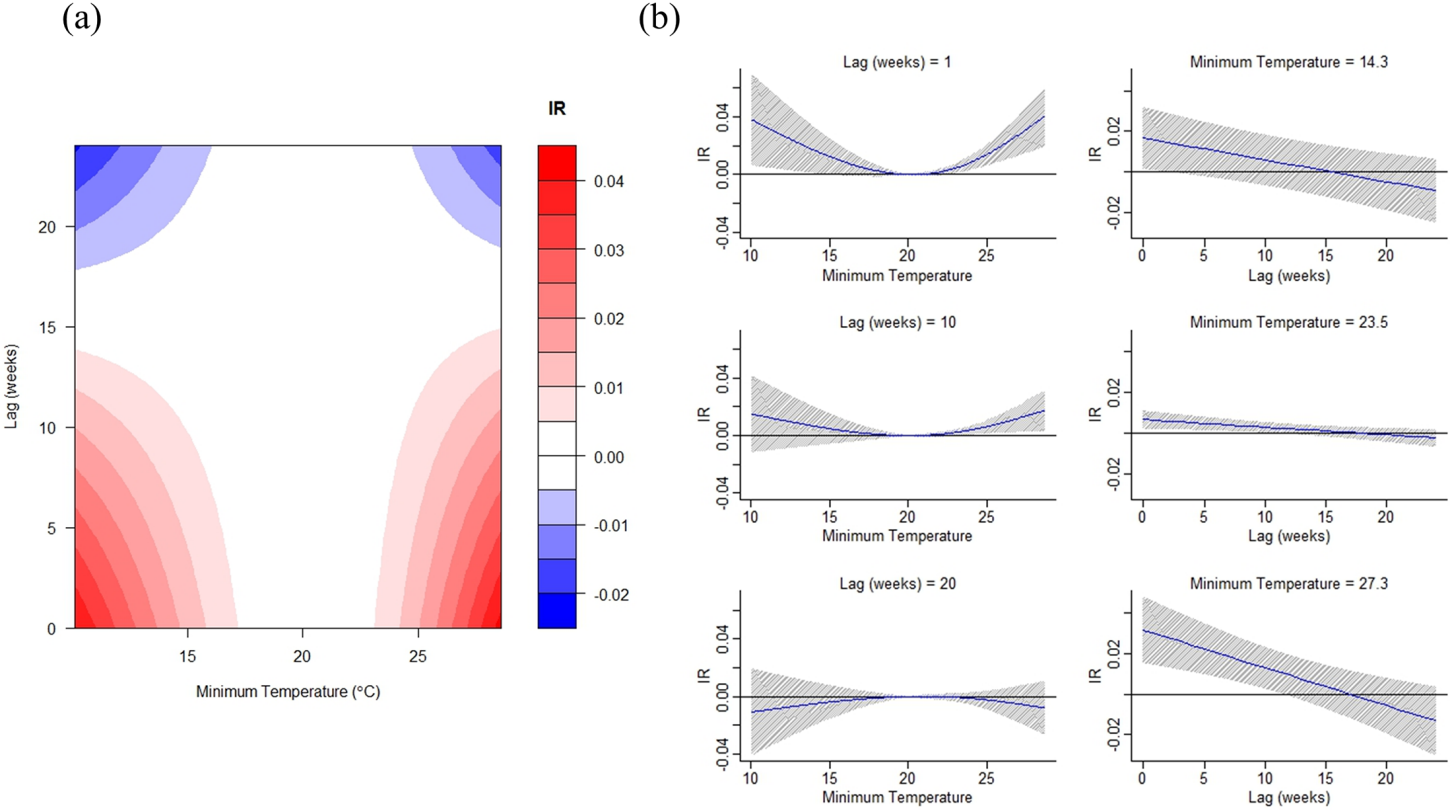
Distributed lag non-linear model results of dengue incidence and weekly average minimum temperature (1998–2012). (a) The contour plot (x-axis indicates the weekly average minimum temperature. y-axis indicates the number of lag weeks. IR indicates the changes in incidence rate (per 100,000) compared with the reference minimum temperature value (mean = 23.4°C). (b) The exposure-response analysis (value: weekly minimum temperature at 5%, 50%, and 95% of its distribution. The grey zone indicates the 95% confidence interval of estimated incidence rate under the specific exposure-response value).

**Fig 4:**
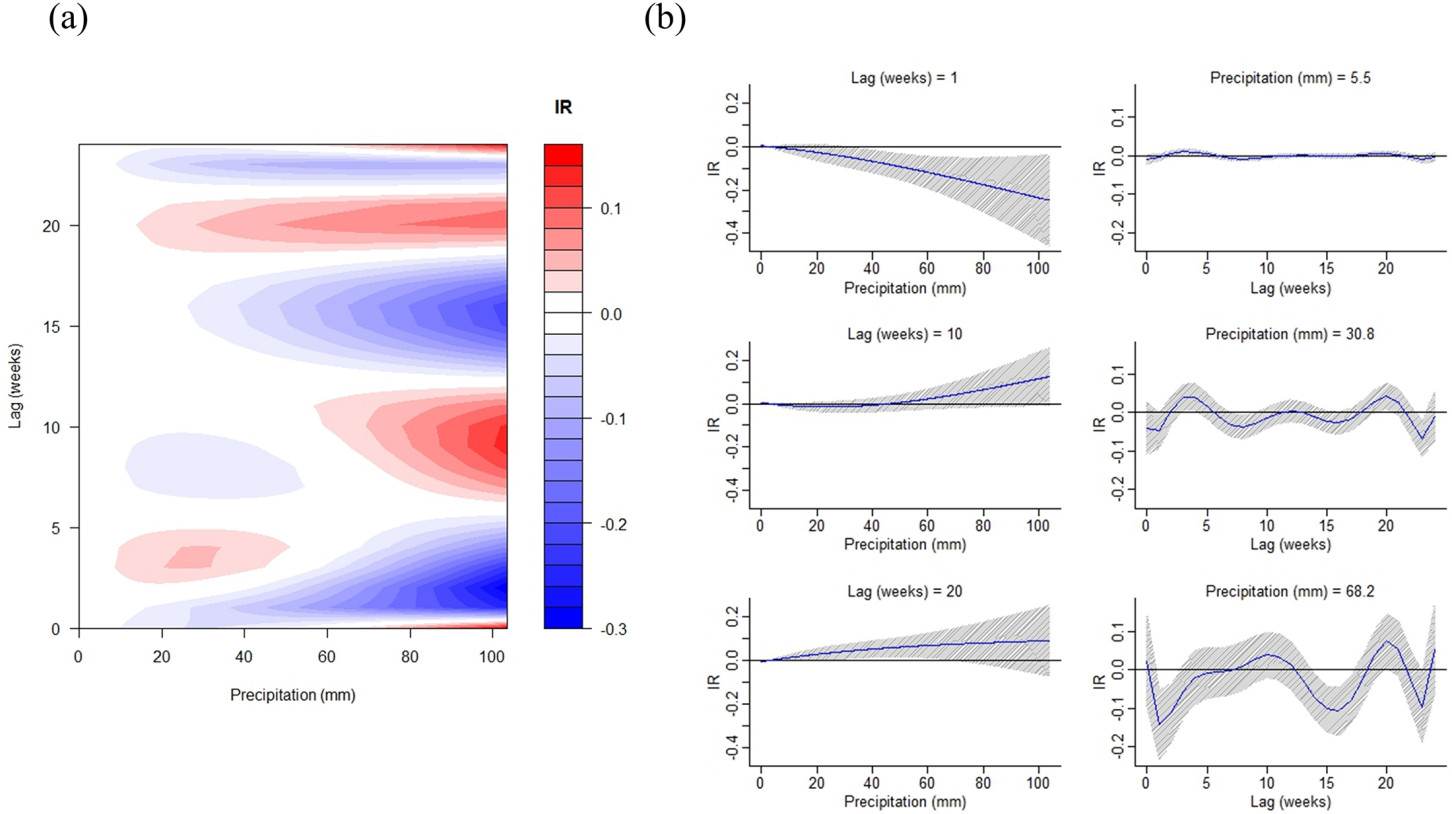
Distributed lag non-linear model results of dengue incidence and weekly average precipitation. (1998–2012). (a) The contour plot (x-axis indicates the weekly average precipitation. y-axis indicates the number of lag weeks. IR indicates the changes in incidence rate (per 100,000) compared with the reference precipitation value (mean = 2.33 mm). (b) The exposure-response analysis (value: weekly average precipitation at 50%, 90%, and 99% of its distribution. The grey zone indicates the 95% confidence interval of estimated incidence rate under the specific exposure-response value).

Weekly minimum temperature and precipitation were included in the DLNM model of best fit (Table 1). The non-linear patterns were obvious for both minimum temperature and rainfall (Figs 3 and 4). Minimum temperature demonstrated bi-modal associations with dengue transmission. Dengue incidence rate increases when the value is either higher than 23°C or lower than 17°C (Fig 3a). The exposure-response analyses indicate that such associations gradually decline to nil around 10 to 15 weeks (Fig 3b). Low to moderate precipitation (20–40 mm) can slightly increase dengue incidence rates within 5 weeks (Fig 4a). Moderate to heavy rainfall has a stronger effect, which might increase transmission risk at lag 10 or 20 weeks. The negative impacts of heavy rainfall on dengue incidence can be observed in either the short term or at around lag 15 weeks (Fig 4b).

**Table 1:** Model selection results based on Akaike’s information criterion*. (lower AIC indicates better model fit).

Variables	AIC	AIC Difference
Null model	151.234	-
Minimum Temperature	150.060	-1.174
Precipitation	147.461	-3.773
Relative Humidity	165.445	14.211
Minimum Temperature + Precipitation**	**137.944**	**-13.291**
Minimum Temperature + Relative Humidity	151.844	0.610
Precipitation + Relative Humidity	149.897	-1.337
Minimum Temperature + Precipitation + Relative Humidity	140.977	-10.258

AIC: Akaike information criterion

* The model is fitted using training data from 1998 to 2012.

** Best-fit model: 2 degrees of freedom for the natural cubic spline for minimum temperature and precipitation. The degree of polynomial function for the lag effects is 1 for minimum temperature and 8 for precipitation. The adjusted-R squared = 0.93.

**Fig 5:**
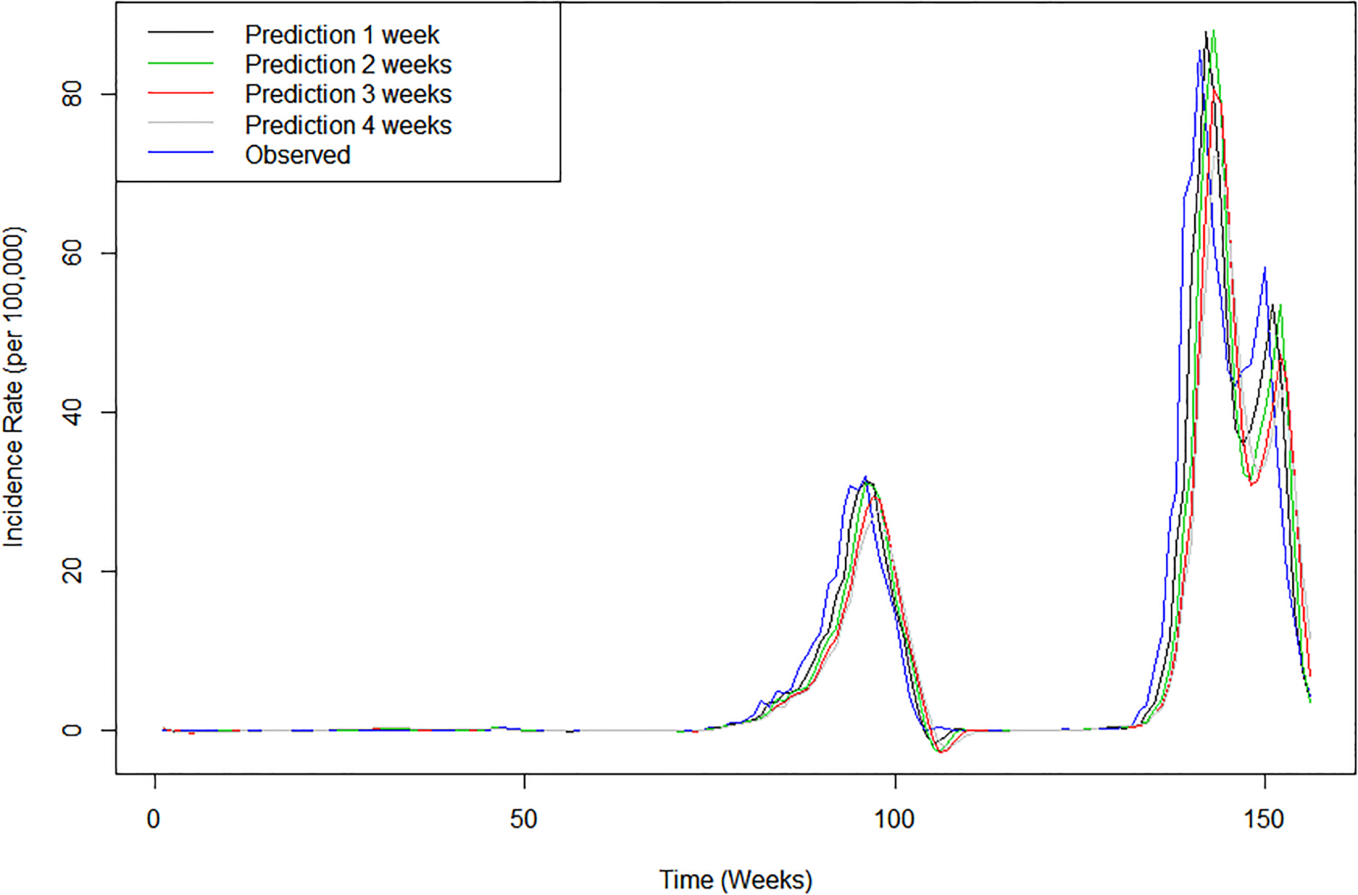
Forecasting results of dengue incidence in 2013, 2014 and 2015 using the best-fit DLNM. (Observed: the weekly incidence rate of dengue fever. Prediction 1 week: forecasted dengue incidence rates one week ahead. Prediction 2 weeks: forecasted dengue incidence rates two weeks ahead. Prediction 3 weeks: forecasted dengue incidence rates three weeks ahead. Prediction 4 weeks: forecasted dengue incidence rates four weeks ahead. x-axis indicates the number weeks in the three forecasted years).

**Fig 6:**
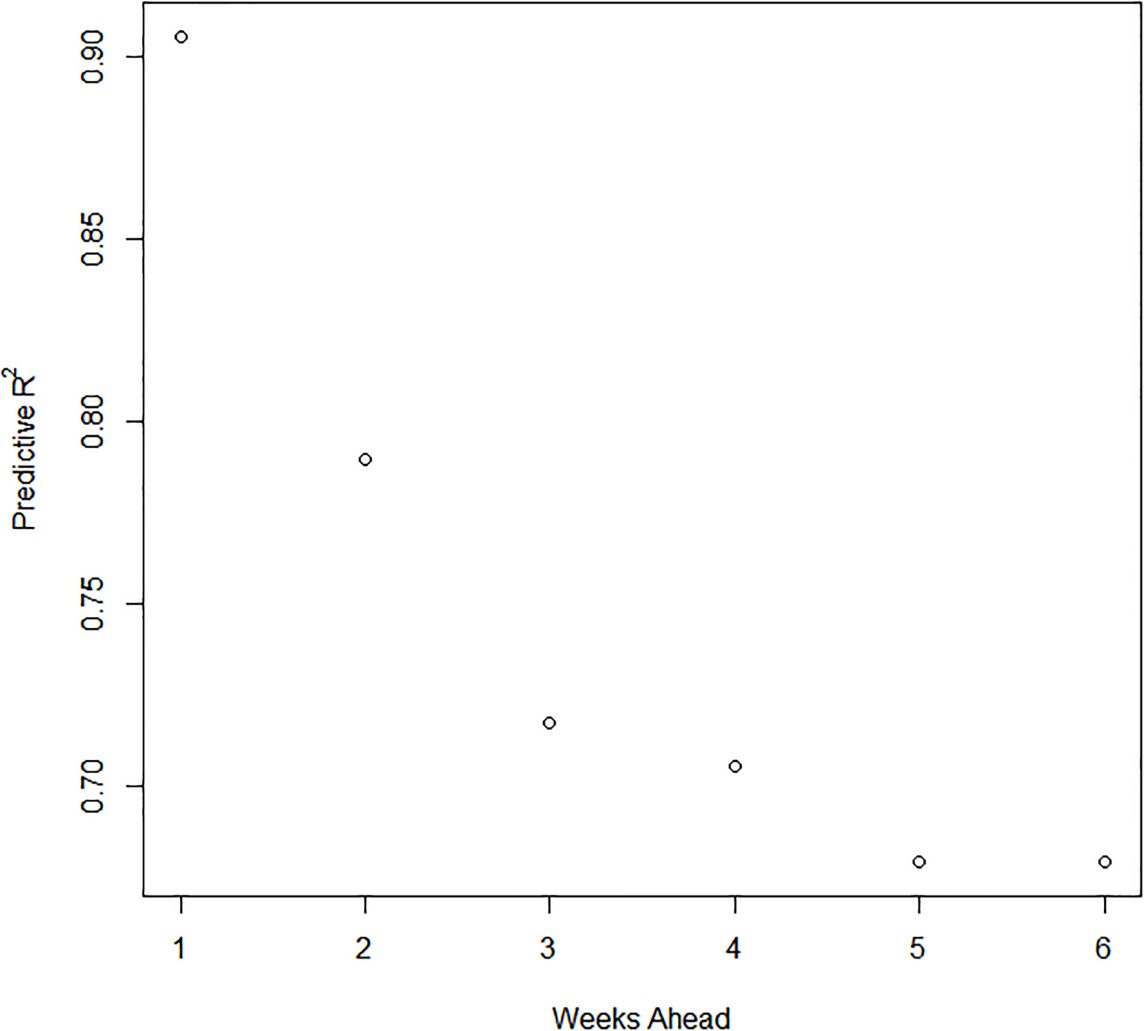
Predictive R-squared. (Weeks ahead indicates the number of weeks predicted ahead by the best-fit model).

The best fitting DLNM model was used to forecast 2013, 2014, and 2015 dengue outbreaks in southern Taiwan (Fig 5). The model produced accurate forecasting results in both high (2014 and 2015) and low transmission (2013) years in predictions for different numbers of weeks ahead. The predictive accuracy was validated by the predictive R-squared (Fig 6). Although accuracy gradually decreased as the number of weeks ahead increased, the values range from 0.9 to 0.7, which indicates a good predictive performance for dengue incidence.

**Fig 7:**
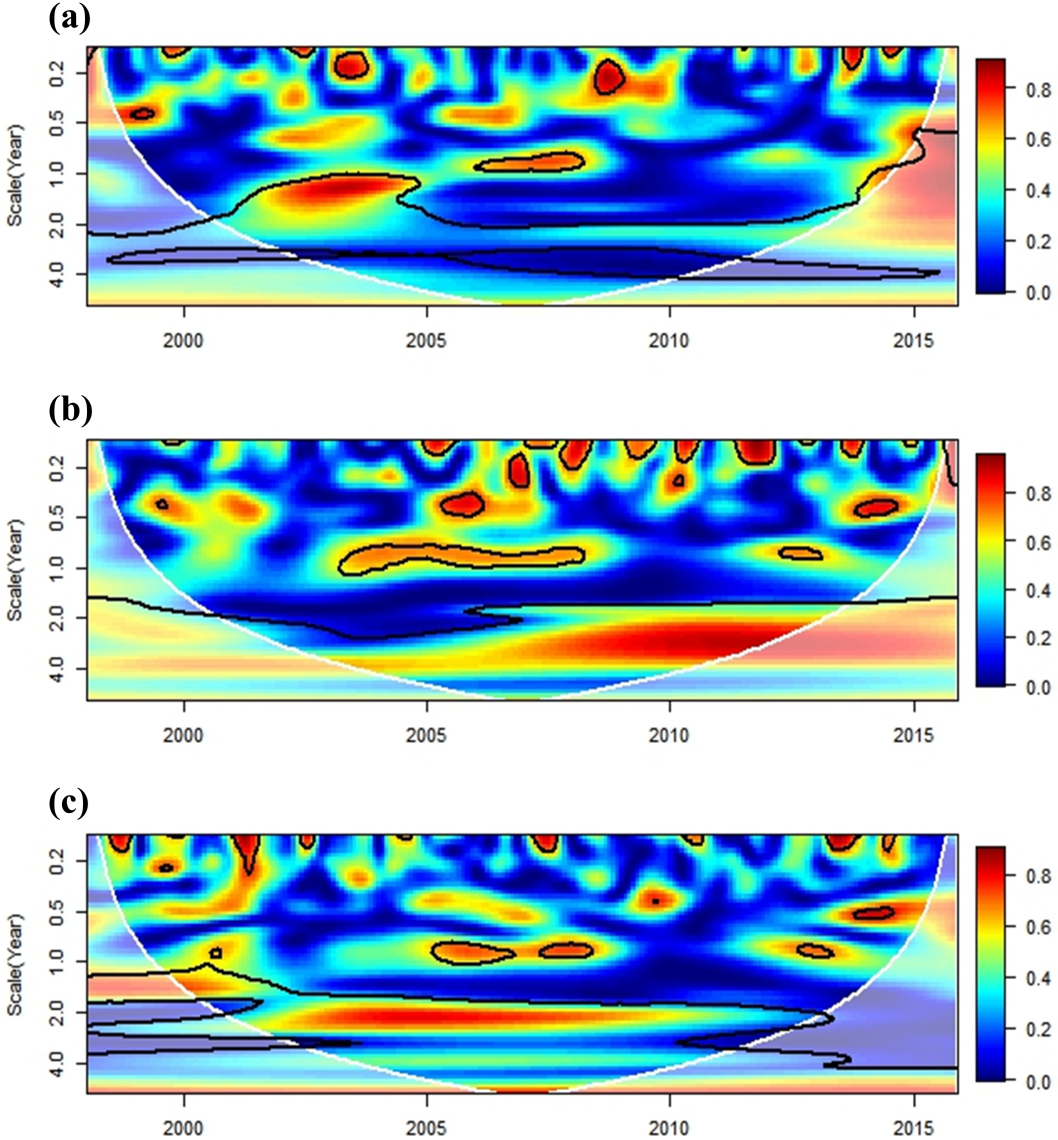
Cross-wavelet coherence analysis of monthly dengue incidence and local climate variables with ENSO (NINO 3.4 index). (a) dengue, (b) minimum temperature, (c) precipitation. The cross-wavelet coherence scale is from 0 (blue) to 1 (red). The cone of influence (results are not influenced by the data edges) and the significantly coherent time-frequency regions (*p* < 0.05) are indicated by solid black lines.

**Fig 8:**
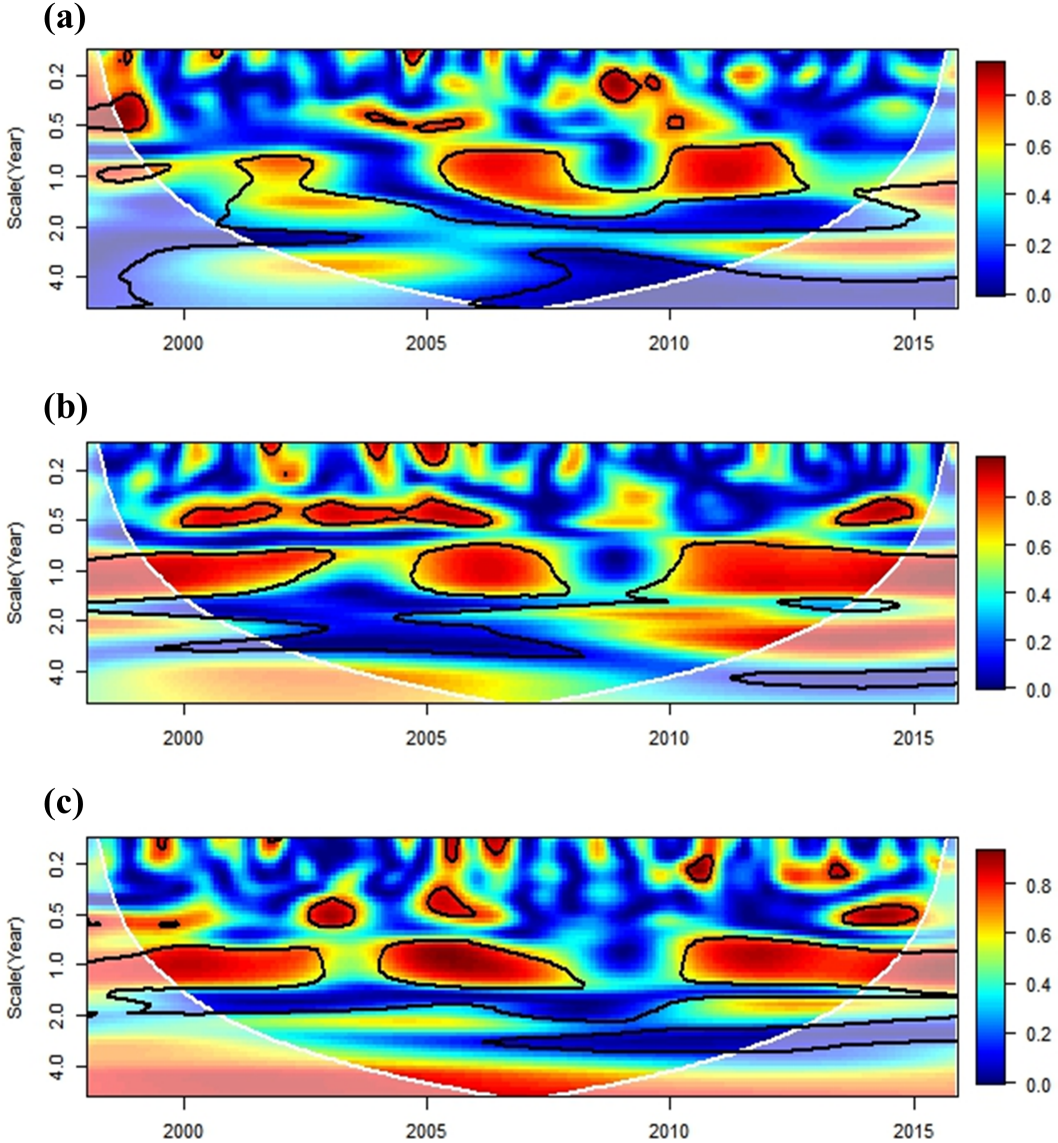
Cross-wavelet coherence analysis of monthly dengue incidence and local climate variables with IOD (Dipole mode index). (a) dengue, (b) minimum temperature, (c) precipitation. The cross-wavelet coherence scale is from 0 (blue) to 1 (red). The cone of influence (results are not influenced by the data edges) and the significantly coherent time-frequency regions (*p* < 0.05) are indicated by solid black lines.

The relationship between regional climate and dengue transmission was investigated using a cross-wavelet coherence analysis. The ENSO exhibited a synchronized pattern with dengue incidence on a seasonal (1-year frequency) scale during 2002–2004, 2006–2007, and 2015. These findings coincide with major historical outbreaks in southern Taiwan (2002, 2007, and 2015), with the exception of the 2014 outbreak (Fig 7a). The ENSO was associated with local minimum temperature on a 1-year scale during 2004–2007 and on a 3-year scale after 2006 (Fig 7b). For precipitation, the coherence pattern indicated a 2-year scale for 2002–2010 (Fig 7c). The association between IOD and dengue has a different pattern to ENSO in that a 1-year scale is significant during 2005–2012 (Fig 8a). IOD had a stronger impact on the seasonality of minimum temperature and precipitation at 1-year scale (Fig 8b and 8c).

## Discussion

Climate is an important trigger of mosquito-borne disease, although the precise relationship varies according to local vector ecology across the geographical range of both vector and disease distributions [5, 10]. Previous studies conducted in Asia and Latin America have revealed the importance of climate conditions in dengue transmission [54–57]. In this study, we found that weekly minimum temperature has a significant bimodal positive effect on dengue transmission when the value is either higher than 23°C or lower than 17°C, and the effects can last for 10–15 weeks. The effect of higher temperatures can shorten the duration of mosquito development and virus propagation, which could be related to rapid vector population growth and higher transmission risk (Fig 3a). Chaves et al. [58] reported a 10-week lag between hot events and *Ae*. *aegypti* population outbreaks, which further supports the hypothesis that climate-affected changes in vector abundance could drive unforeseen dengue epidemics. We also identified a correlation between a low minimum temperature and higher dengue incidence. This is usually between December and February, when the weekly minimum temperature is below 17°C in southern Taiwan. Thus, the effect of low temperature might be related to dengue transmission in winter. However, dengue is not endemic in Taiwan and the epidemic season usually starts in May or June (Fig 1d). The dengue cases identified in winter are the residual of previous outbreaks. We hypothesize that colder temperatures might force mosquitos and people to stay indoors, which could increase the probability of contact and the risk of infection. Previous studies also demonstrated that low mean temperature (< 18°C) might be associated with shorter EIP and potential higher dengue transmission risk when the diurnal temperature range (DTR) is large (20°C) [59, 60]. However, it’s very rare to have such large DTR in southern Taiwan so the effect of low minimum temperature requires further investigation.

Unlike temperature, precipitation tends to have inconsistent effects across different studies [33, 37]. Both the timing and amount of precipitation are crucial for dengue transmission, and this study showed that higher precipitation can increase dengue incidence in specific temporal windows (lag = 10 weeks or lag = 20 weeks). In contrast, extremely high precipitation could have a washout effect in the short term, thus reducing mosquito survival and the risk of transmission. However, low to moderate rainfall can still slightly increase dengue incidence in the short term (lag = 0–5 weeks) (Fig 5a). Precipitation can create potential habitats for the aquatic stage of mosquito development and affect their distribution. The non-linear effects of precipitation have been shown in many studies [11, 35, 61]. In Singapore, high precipitation also had a positive effect on dengue transmission at a lag of 5–20 weeks and a negative impact at lag of 1–4 weeks [62]. A similar study conducted in Mexico demonstrated that higher precipitation increases dengue transmission as long as monthly precipitation remains below 550 mm [11]. Although rainfall is thought to produce breeding habitats for mosquitos, this scenario may apply to a greater extent to *Ae*. *albopictus* rather than *Ae*. *aegypti*, which prefer to live indoors [18]. In southern Taiwan, droughts sometimes occur, and people tend to use containers to store water temporarily during the dry season, which may increase mosquito survival and the likelihood of human contact. Furthermore, southern Taiwan is located in a tropical region and frequently experiences summertime typhoons, which bring extreme rainfall and strong winds [37]. These situations make it difficult to evaluate rainfall in a linear manner, which is why the DLNM provides a better approach to understanding the influence of precipitation.

Chien et al. [35] have applied a similar model to evaluate the effect of weather on dengue transmission in southern Taiwan; however, the study emphasized the evaluation of spatial variations, rather than predicting future outbreaks. The dengue prediction model developed in our study demonstrated good predictive performance for dengue incidence in the low transmission year (2013) and the high transmission years (2014 and 2015) after controlling the seasonality and autocorrelation. The predictive accuracy is still good (predictive R-squared = 0.7) when predicting dengue incidence 6 weeks ahead. The current dengue control strategies in Taiwan, which include vector control with insecticides and source reduction, as well as community education, are initiated after identifying the first local human case. This “delayed” time frame of disease prevention results in the uncontrolled propagation of the virus at the early stage, which can be heavily magnified when vector populations are also unchecked. Thus, the 6-week prediction might inform public health authorities for an early implementation of control measurements in southern Taiwan, especially in the hotspot areas. Forecasts also provide a benchmark against which it is possible to compare the course of the epidemic, evaluating how effective have been control measures when comparing forecasts with observed cases. Thus models like the one presented here could provide important information for decision makers in charge of dengue prevention in Taiwan.

The study evaluated the influences of both IOD and ENSO on dengue transmission in the study area. The effects of ENSO exhibited patterns that were synchronized with the major dengue outbreaks in southern Taiwan (2002, 2007, and 2015), and IOD could be more relevant to inter-annual epidemic patterns. IOD also had a stronger impact on the seasonality of local climate conditions than the ENSO effect. It is worth mentioning that the influence of ENSO on minimum temperature became significant after 2006 (3-year scale), which could be related to the most recent dengue epidemics. The effects of regional climate phenomena on dengue transmission also varied across different study areas. Tipayamongkholgul et al. [19] indicated that ENSO explains 15% to 22% of monthly dengue incidence in Thailand. In Pakistan, a dengue outbreak in 2011 was linked to IOD, and ENSO could be responsible for more recent epidemics [42]. In Bangladesh, only IOD exhibited synchronized patterns with the dengue incidence [63]. In contrast, Descloux et al. [64] did not find any evidence of an ENSO effect when they analyzed the effects of local and regional meteorological variables on dengue incidence in Noumea-New Caledonia (western Pacific Ocean). Our study showed that regional climate phenomena could affect local climate variations and dengue transmission in Taiwan. The influence of ENSO or IOD should be considered in future work on epidemic forecasting.

The unprecedented dengue outbreaks in southern Taiwan during 2014 and 2015 represented a significant burden for public health infrastructure and clinical services. However, the context surrounding the two years were not identical, as there was a petrochemical gas explosion in Kaohsiung City in late-July 2014. The explosion damaged buildings, roads, and drainage systems in dengue-hotspot areas. Following the high temperatures and heavy rainfall in August, the number of dengue cases increased sharply. The accident could have been a catalyst in the dengue outbreak because the surface damage and heavy rainfall could have facilitated the growth of the mosquito population. The residents have been relocated to a temporary refuge, which could have increased the possibility of being exposed to mosquito vectors. The accident accompanied with climate fluctuations could have altered the dynamics of transmission between the vector and host, and affected the subsequent outbreak [65]. In addition, NS1 antigen detection has been used as a rapid diagnostic technique since 2014, which could also have helped local physicians to detect and report more dengue cases.

Although we have developed a useful model to predict dengue incidence using climate parameters, there are a couple of limitations in this study. Climate variability is not the only risk factor for dengue transmission. Our model does not explicitly consider the impacts of secondary transmission and herd immunity in seasonal dengue outbreaks. However, dengue fever is not endemic in Taiwan. Thus, herd immunity might not play a significant role in shaping dengue transmission. Secondary transmission is also a complex phenomenon, which could be related to virus serotype, vector control, movement of people, and exposure to infectious mosquito bites [66]. Addressing these issues requires more complicated mathematical/statistical models capable of simulating various parameters that were not included in this study.

Another limitation is that our model did not include vector data. Chang et.al. [67] developed models to include different vector indices along with meteorological variables in the high-risk dengue district of Taiwan. The results indicated that the model’s performance can be improved if vector data is included. In Singapore, the mosquito abundance index is the most important parameter for forecasting dengue incidence using the least absolute shrinkage and selection operator (LASSO) approach [25]. A similar technique can be used in Taiwan if vector data is available. Unfortunately, there is no standardized mosquito surveillance system in southern Taiwan. Such a system needs to be put in place to help researchers to understand local vector ecology and dengue transmission. This would also make it possible to verify the direct impact of climate on vector populations. Despite these limitations, our climate-based dengue forecasting model achieved good prediction performance using the data in the dengue non-endemic country. The model might be useful to be implemented in regions with similar dengue epidemiology to southern Taiwan. These type of models might also be used to forecast the risk of Zika or Chikungunya virus which have the same vector and similar geographical epidemic areas.

The magnitude of dengue incidence in 2014 and 2015 represented a huge public health burden in southern Taiwan. This study developed a useful predictive model for a dengue early warning system, which can provide reliable forecasting results 6 weeks ahead. The timeframe would allow public health decision-makers to initiate interventions and allocate resources for disease control/prevention. We also highlighted the importance of considering both regional and local climate fluctuations, which could have an unusual impact on dengue transmission in terms of future climate change.

## Supporting information

S1 Fig Cross-wavelet coherence analysis of monthly dengue incidence rates and local climate variables with different ENSO indices. (a-c) Multivariate ENSO Index (MEI) vs. dengue, minimum temperature, and precipitation. (d-f) NINO 1+2 Index vs. dengue, minimum temperature, and precipitation. (g-i) NINO 3 Index vs. dengue, minimum temperature, and precipitation. (j-l) NINO 4 Index vs. dengue, minimum temperature, and precipitation. The cross-wavelet coherence scale is from 0 (blue) to 1 (red). The cone of influence (results are not influenced by the data edges) and the significantly coherent time-frequency regions (*p* < 0.05) are indicated by solid black lines.
(TIF)

S2 Fig Autocorrelation function (ACF) and partial autocorrelation function (PACF) of dengue time-series data from 1998 to 2015. The dotted blue lines indicate a significant level. The lag refers to the number of weeks.
(TIF)

S3 Fig Autocorrelation function (ACF) and partial autocorrelation function (PACF) of the residual. The autoregressive terms (lag = 5) were included to adjust for seasonality and autocorrelation in the model. The dotted blue lines indicate a significant level. The lag refers to the number of weeks.
(TIFF)

S1 Table Model comparison results of seasonal adjustment. (PDF)

S2 Table Annual meteorological parameters in southern Taiwan from 1998 to 2015. (PDF)
