# Correction: Effects of local and regional climatic fluctuations on dengue outbreaks in southern Taiwan

**DOI:** 10.1371/journal.pone.0181638

**Published:** 2017-07-13

**Authors:** The PLOS ONE Staff

Previous versions of the Supporting Information files S1, S2 and S3 Figs, S1 and S2 Tables were published in error. The publisher apologizes for the errors. Please see the correct Supporting Information files below.

## Supporting information

S1 Fig Cross-wavelet coherence analysis of monthly dengue incidence rates and local climate variables with different ENSO indices. (a-c) Multivariate ENSO Index (MEI) vs. dengue, minimum temperature, and precipitation. (d-f) NINO 1+2 Index vs. dengue, minimum temperature, and precipitation. (g-i) NINO 3 Index vs. dengue, minimum temperature, and precipitation. (j-l) NINO 4 Index vs. dengue, minimum temperature, and precipitation. The cross-wavelet coherence scale is from 0 (blue) to 1 (red). The cone of influence (results are not influenced by the data edges) and the significantly coherent time-frequency regions (*p* < 0.05) are indicated by solid black lines.
(TIF)

S2 Fig Autocorrelation function (ACF) and partial autocorrelation function (PACF) of dengue time-series data from 1998 to 2015. The dotted blue lines indicate a significant level. The lag refers to the number of weeks.
(JPEG)

S3 Fig Autocorrelation function (ACF) and partial autocorrelation function (PACF) of the residual. The autoregressive terms (lag = 5) were included to adjust for seasonality and autocorrelation in the model. The dotted blue lines indicate a significant level. The lag refers to the number of weeks.
(TIFF)

S1 Table Model comparison results of seasonal adjustment. (PDF)

S2 Table Annual meteorological parameters in southern Taiwan from 1998 to 2015. (PDF)
