# Supplementary figures and images for: Effects of local and regional climatic fluctuations on dengue outbreaks in southern Taiwan
Source: PLoS One. 2017 Jun 2;12(6):e0178698. doi: 10.1371/journal.pone.0178698 (PMC5456348; doi:10.1371/journal.pone.0178698)

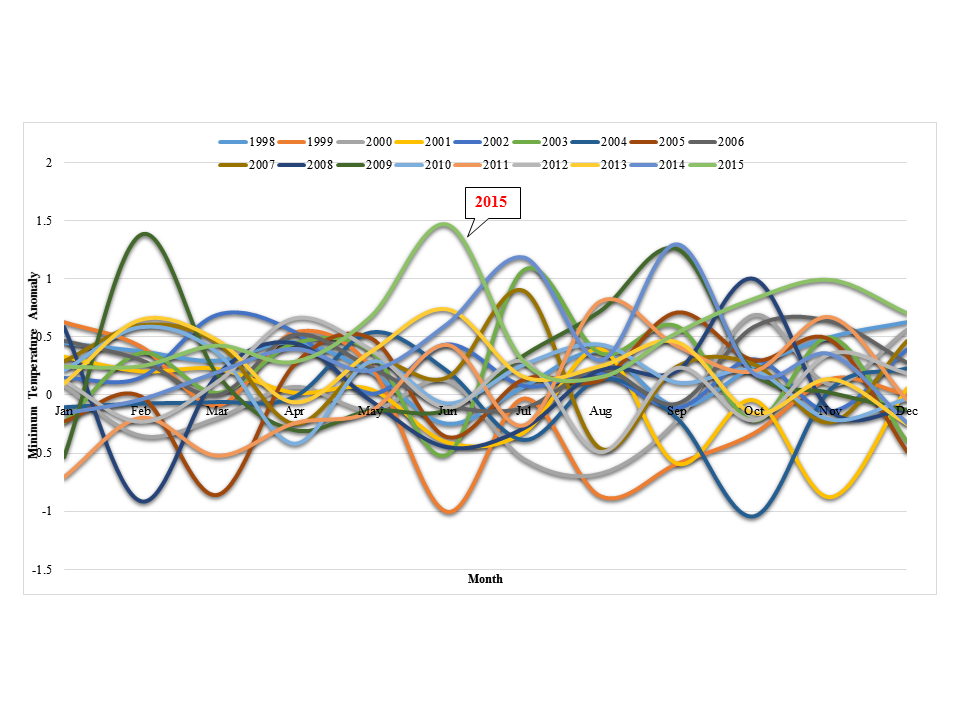

Supplement: S1 Fig — (a-c) Multivariate ENSO Index (MEI) vs. dengue, minimum temperature, and precipitation. (d-f) NINO 1+2 Index vs. dengue, minimum temperature, and precipitation. (g-i) NINO 3 Index vs. dengue, minimum temperature, and precipitation. (j-l) NINO 4 Index vs. dengue, minimum temperature, and precipitation. The cross-wavelet coherence scale is from 0 (blue) to 1 (red). The cone of influence (results are not influenced by the data edges) and the significantly coherent time-frequency regions (p < 0.05) are indicated by solid black lines. (TIF) [file pone.0178698.s001.tif]

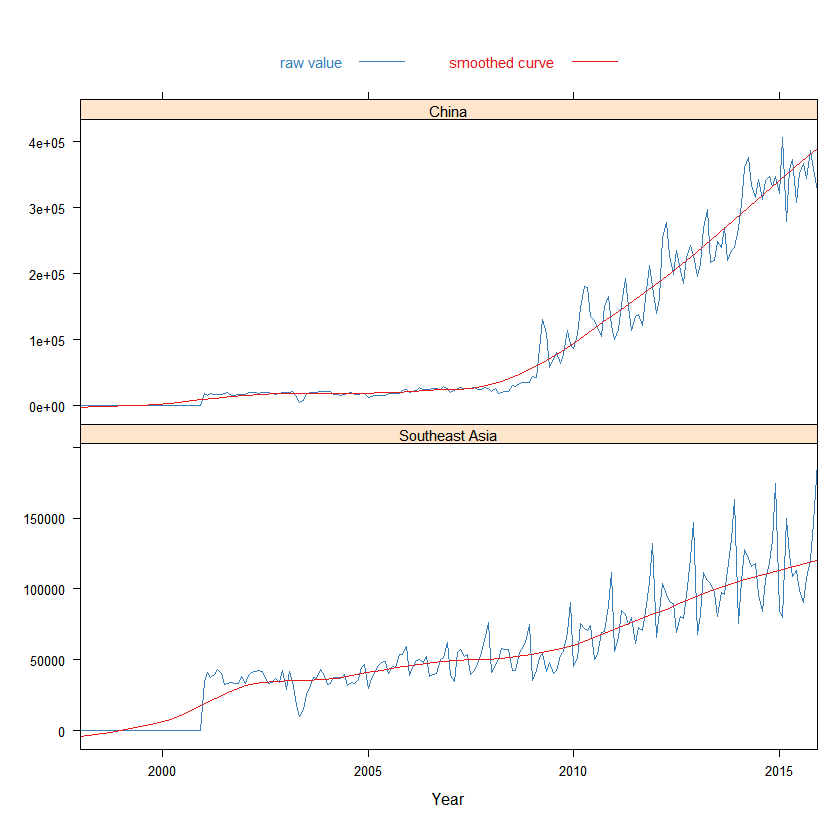

Supplement: S2 Fig — The dotted blue lines indicate a significant level. The lag refers to the number of weeks. (TIF) [file pone.0178698.s002.tif]

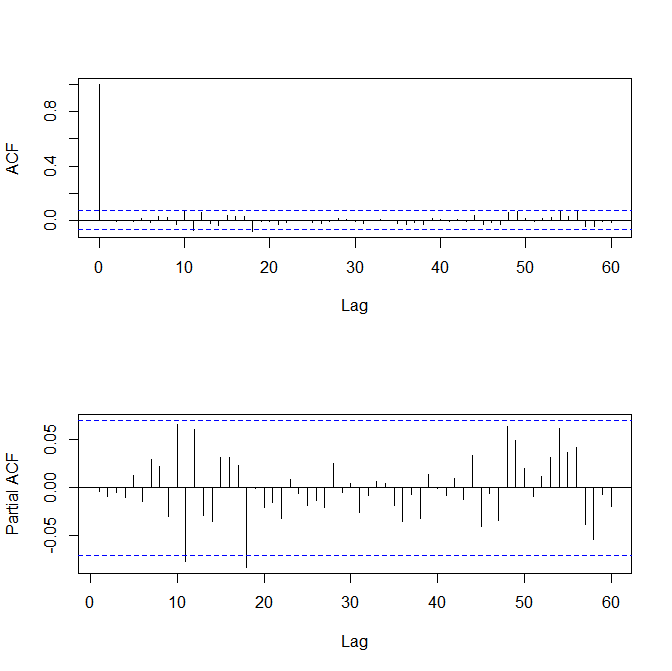

Supplement: S3 Fig — The autoregressive terms (lag = 5) were included to adjust for seasonality and autocorrelation in the model. The dotted blue lines indicate a significant level. The lag refers to the number of weeks. (TIFF) [file pone.0178698.s003.tiff]
