# Supplementary material for: Effects of local and regional climatic fluctuations on dengue outbreaks in southern Taiwan
Source: PLoS One. 2017 Jun 2;12(6):e0178698. doi: 10.1371/journal.pone.0178698 (PMC5456348; doi:10.1371/journal.pone.0178698)
Supplement: S2 Table — (PDF) [file pone.0178698.s005.pdf]

S2 Table. Annual meteorological parameters in southern Taiwan from 1998 to 2015.

Year	Meteorological parameters									
	TMEAN	SD	TMIN	SD	TMAX	SD	PREC	SD	RH	SD
1998	25.45	3.94	22.43	4.07	29.44	3.82	5.85	17.03	78.14	5.42
1999	24.70	3.99	21.69	4.25	28.78	3.77	6.71	20.28	77.97	5.89
2000	24.76	4.04	21.81	4.16	28.92	3.82	4.54	14.85	79.36	5.67
2001	24.83	3.99	21.71	4.38	29.16	3.73	6.39	25.18	77.43	6.67
2002	25.28	3.93	22.31	4.31	29.37	3.62	3.08	10.55	74.77	6.23
2003	25.12	4.27	22.10	4.74	29.22	3.88	3.05	16.07	75.62	6.47
2004	24.92	4.27	21.69	4.52	**29.46**	4.15	3.48	15.43	74.86	6.27
2005	24.82	4.85	21.73	5.22	29.13	4.64	**8.18**	29.21	75.54	7.54
2006	25.34	3.99	22.36	4.24	29.45	3.76	5.36	21.41	78.26	6.75
2007	25.21	4.02	22.27	4.33	29.23	3.73	6.03	22.98	75.31	6.83
2008	24.85	4.41	21.89	4.73	28.89	4.15	6.20	24.77	74.05	6.83
2009	25.10	4.27	22.17	4.66	29.13	3.86	4.28	29.59	73.69	5.68
2010	25.05	4.21	22.07	4.58	29.10	3.93	5.40	21.03	72.78	6.10
2011	24.48	4.69	21.72	4.95	28.32	4.46	4.13	16.21	73.53	6.17
2012	24.91	4.22	22.04	4.46	28.81	4.02	6.31	19.30	76.07	6.72
2013	25.05	4.24	22.23	4.54	28.74	3.90	4.63	19.62	73.98	6.73
2014	25.13	4.55	22.30	5.00	28.72	4.26	4.40	17.96	73.94	6.37
2015	**25.55**	4.08	**22.88**	4.44	29.14	3.65	3.87	13.64	74.68	6.55
Average	25.03	4.22	22.08	4.53	29.06	3.95	5.10	19.73	75.55	6.38

TMEAN=mean temperature, SD=standard deviation, TMIN=minimum temperature, TMAX=maximum temperature, PREC=precipitation, RH= relative humidity. The maximum value for each parameter is shown in bold.
