# Supplementary material for: Correction: Effects of local and regional climatic fluctuations on dengue outbreaks in southern Taiwan
Source: PLoS One. 2017 Jul 13;12(7):e0181638. doi: 10.1371/journal.pone.0181638 (PMC5509367; doi:10.1371/journal.pone.0181638)
Supplement: S1 Table — (PDF) [file pone.0181638.s004.pdf]

S1 Table. Model comparison results of seasonal adjustment.

Seasonality Adjustment	AIC
Sine (frequency: 54 weeks)*	1961.957
Cosine (frequency: 41 weeks)	2046.496
Sine + Cosine (frequency: 54 weeks)	2047.474
Natural cubic spline (degree of freedom: 6)**	1941.953
Autoregressive term (lag=1 week)	255.861
Autoregressive term (lag=2 weeks)	241.757
Autoregressive term (lag=3 weeks)	231.188
Autoregressive term (lag=4 weeks)	156.768
**Autoregressive term (lag=5 weeks)**	**151.243**

*: The frequency of week was determined by comparing the AIC. (Range:1-60 weeks).

**: The degree of freedom was determined by comparing the AIC. (Range: 1-9).
